# Exploring the Link between Oil Exploitation and Cancer in the Indigenous Population of Ecuador: A Scoping Review

**DOI:** 10.3390/ijerph19052674

**Published:** 2022-02-25

**Authors:** Tibo Uyttersprot, François Janssens, Danielle Fernandes, Wei-Hong Zhang

**Affiliations:** 1 Department of Public Health and Primary Care, Faculty of Medicine and Health Sciences, Ghent University, 9000 Ghent, Belgium; tibo.uyttersprot@ugent.be (T.U.); danielle.fernandes@ugent.be (D.F.); weihong.zhang@ugent.be (W.-H.Z.); 2 International Centre for Reproductive Health (ICRH), Ghent University, 9000 Ghent, Belgium; 3 School of Public Health, Université Libre de Bruxelles (ULB), 1070 Brussels, Belgium

**Keywords:** cancer, oil exploitation, indigenous, Ecuador, environmental health, health policy

## Abstract

With cancer accounting for 19% of deaths and projected to rise in the coming years, Ecuador’s inequities in healthcare coverage remain a major concern for the rural, indigenous populations. While the cancer burden among this vulnerable population has been much publicized in the context of the controversial oil extraction in the Amazon, there is contradictory evidence on its occurrence and determinants. This review critically discusses the available literature on cancer among indigenous people in Ecuador and explores the link between oil exploitation and cancer occurrence among indigenous people using a scoping review approach. The results of this review show there is a clear but inconsistent association between oil exposure and cancer risk in indigenous populations of Ecuador. While the environmental magnitude of oil extraction in this region is a topic of debate, our findings point to the interplay with social determinants and other sources of carcinogenic compounds, which exacerbates the risks faced by indigenous communities. Based on these findings, this study puts forward three arguments to contextualize the occurrence of cancer related to oil exploitation in the Amazon, and puts forth key recommendations for public health initiatives embedded within the local community.

## 1. Introduction

Non-Communicable Diseases (NCDs) like cancer, diabetes, cardiovascular diseases, and chronic respiratory diseases, which account for 71% of total deaths worldwide, are increasingly posing a major health challenge [1]. Among these, cancer features prominently as the second leading cause of death globally, with its case fatality rate projected to increase steadily in the next decade [2]. While once only considered a burden for High-Income-Countries (HICs), developing countries are rapidly closing this gap. They experience a change in lifestyle patterns similar to the developed world, with increased exposure to carcinogens, bad dietary practices, and sedentary occupations [3]. This shift in disease ecologies proves to be a significant concern, with Low- and Middle-Income Countries (LMICs) accounting for approximately 78% of the global deaths due to NCDs, and 65% of all cancer deaths [1,4]. Furthermore, alarming statistics on the inadequate coverage of cancer screening and diagnostic and treatment services in most LMICs demonstrate that these countries are ill-equipped to cope with the mounting health challenge [5,6].

Similar to other developing countries in Latin America, Ecuador has been experiencing such a change in disease profiles precipitated by socio-economic changes in the past few decades [7]. In the latter part of the twentieth century, Ecuador experienced an economic boom as powerful multinational companies descended on the oil-rich Amazon region and allowed colonizers to initiate commercial farming [8]. Several other Latin American countries experienced similar systemic extraction of their natural resources by foreign concerns, hitching their fortune and the future of their people to oil production. Ecuador, Mexico, and Brazil are major global exporters of oil, while Chile and Peru are some of the top global producers of tin and copper [9]. These extractive activities make their way to remote, resource-rich lands, often inhabited by indigenous people. Such extractive development projects on indigenous land are fraught with complications particularly due to the tenuous and complex relationship between indigenous communities and state systems. Many indigenous groups were traditionally excluded from the wider national society and some were involuntarily incorporated into the state system [10,11]. The struggles of the Mapuche people in Chile and the conflict between Vale do Rio Duce and the Tucuma indigenous communities in Brazil bear testament to this struggle [12,13]. One of the most visible indigenous movements against oil extraction is led by Ecuador’s indigenous peoples. Ecuador exemplifies the phenomenon of a ‘resource curse’ where, despite its natural resource abundance, the expansion of extractive activities has compromised social, environmental, and health concerns [9,14]. Prompting increased urbanization, occupational shifts, and lifestyle changes, this socio-economic development has been accompanied by an epidemiological transition, with NCDs accounting for 72% of all deaths, of which 19% are due to cancer [15,16,17]. In turn, this rising health burden has outpaced the nation’s public healthcare, which has a limited number of cancer registries covering just one eighth of the population as of 2017 [18]. Even as Ecuador put in place a new national cancer plan, inequalities in healthcare coverage and limited availability of cancer data remain a concerning and largely unaddressed issue, leaving rural populations most vulnerable [19,20].

Rural Ecuador is largely inhabited by its indigenous population, which comprises more than a million (~7%) of its total population [21,22,23]. Indigenous groups, being largely dependent on natural resources for their livelihoods and sustenance, witnessed alarming changes due to Ecuador’s economic development. The advent of commercial oil extraction in the Ecuadorian Amazon transformed their physical and social environment [24]. While some developments like the expansion of road infrastructure and employment opportunities were welcomed by local communities others have had more far-reaching insidious consequences for their welfare [25]. Indigenous people have been in a bitter decade-long lawsuit with the multinational oil company Chevron, whose exploitative oil extraction was linked to environmental damage and health concerns such as cancer in the surrounding areas [26].

While concerns about the rising occurrence of cancer among indigenous populations have often been cited in news reports, the research evidence has been insufficient and often contradictory [27]. Along with inconclusive reporting on its occurrence, cancer has been attributed to widely different factors in various research studies. Understanding the burden of cancer and the factors relevant to its occurrence is instrumental when advocating and planning for health policies.

Therefore, this paper aims to review and critically discuss the available literature on cancer among indigenous people in Ecuador and explore the link between oil exploitation and cancer occurrence among indigenous people. Based on our findings, we provide key recommendations for public health and social innovations.

## 2. Materials and Methods

As the topic of this study is very under-researched, with highly heterogeneous studies and authors with a conflict of interest, a scoping review exploring the cancer burden in indigenous Ecuadorians and its link to oil exploitation was deemed the most valuable study approach. The research objectives, inclusion criteria and methodological techniques were determined before the study commencement using the Joanna Briggs Institute (JBI) Reviewers’ manual 2015 Methodology for JBI Scoping Reviews and the PRISMA Extension for scoping reviews [28,29].

### 2.1. Research Question

The Population, Concept, and Context (PCC-) approach of the JBI was used for this scoping review. The research question at the centre of our study was the following: What is the occurrence of cancer in the indigenous Ecuadorian population and what are the social and environmental determinants linked to oil exploitation. The population for this review includes the indigenous Ecuadorian people. The concept under exploration is the cancer burden in the predefined population, and the context relates to the social and environmental determinants linked to oil exploitation. The choice for Ecuador as the study setting serves a strategic purpose. First, there have been some local research efforts providing a foundation to expand on. Second, the lack in cancer monitoring undermines the attempts in the battle against cancer, with an amplified impact on the indigenous Ecuadorians [20,30]. Third, the double standards surrounding environmental protection in the Ecuadorian oil extraction industry highlight the necessity for new evidence that can hold oil companies and policymakers accountable for the harm inflicted [31]. A review consolidating all evidence in the Ecuadorian setting could spark a new wind, foster knowledge translation in Ecuador and stimulate other researchers, inside and outside of Ecuador to build further on the topic.

### 2.2. Eligibility Criteria

Peer-reviewed journal articles were included if they were written in English, focused on indigenous people of Ecuador, and described oil extraction-related determinants of cancer among this population (inclusion and exclusion criteria described in Table 1). Papers were excluded if they did not fit into the conceptual framework of the study, published in languages other than English or if they focused on cancer determinants that are not related to oil extraction in the Amazon. Papers that did not meet the eligibility criteria, but described related cancer determinants among the population, were referenced in the discussion section of this review. Considering the limited literature on this topic in the Ecuadorian context, no restrictions were set regarding the year of publication.

### 2.3. Search Strategy

PubMed and Web of Science were consulted as databases in November 2021. The last search was conducted on 10 November 2021.

PubMed and Web of Science were the main search engines and a sensitive search strategy, not including the social and environmental determinants, was used to retrieve all relevant articles. Google Scholar was consulted after searching PubMed and Web of Science to ensure the exhaustiveness of the search.

The identified key words and MeSH terms led to the search strategies in the different online libraries that are summarized in Table 2. Endnote provided a central base to secure the titles and abstracts of the articles and to allow for deduplication.

### 2.4. Study Selection and Information Extraction

The search results were primarily screened on title and abstract and subsequently on full text, using the inclusion and exclusion criteria. The minimal requirement for the articles to be included was to contain information on the cancer burden and/or its link with oil exploitation in the indigenous Ecuadorian population. The articles that passed the first screening on title and abstract fulfilled all inclusion criteria and matched none of the exclusion criteria. In case of disagreement or doubt, articles were re-evaluated during the second screening. This second screening on full text was, just like the first screening on title and abstract, independently conducted by two researchers (FJ, TU). In case of disagreement, a third researcher (DF) was consulted. The 7 articles that passed the second screening on full text fulfilled all inclusion criteria and matched none of the exclusion criteria. Furthermore, forward and backward snowballing was conducted to identify relevant studies from the articles included after full-text screening.

In what follows, evidence on the occurrence of cancer in the indigenous Ecuadorian population and its link with social and environmental determinants related to oil exploitation will be depicted in the results and critically interpreted in the discussion. The extraction table in Appendix A contains a summary of the included studies, with details on the study design and population, type of cancer, and the cancer burden and its main determinants. As this study aims to grasp the scientific landscape on the topic, no risk of bias will be assessed, but the confounders and conflicts of interest of the individual studies will also be reported in the extraction table.

## 3. Results

**Figure 1:**
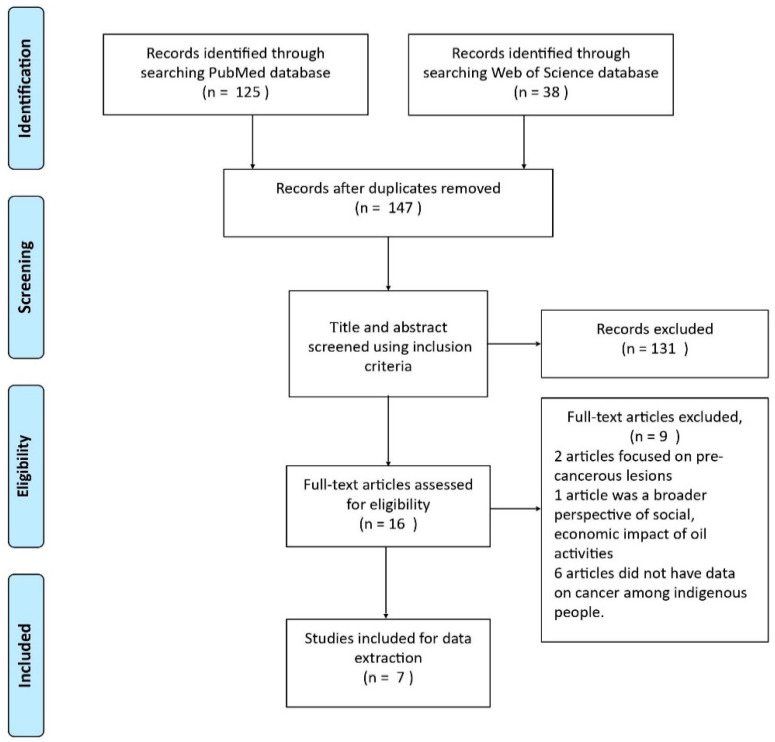
PRISMA flow chart for selection of studies.

A total of 147 articles were returned from searches of the electronic databases, after removing duplicates. Based on a primary screening of the title and abstract, 131 articles were excluded, and 16 eligible articles were selected for full-text screening. Searching for additional articles over Google Scholar and using the forward and backward snowballing method did not elicit any further relevant articles, thus confirming the exhaustiveness of the search strategy on the two main search engines. After screening the 16 full-text articles, two were excluded because they investigated precancerous lesions rather than actual cancer burden and one was excluded as it is a phenomenological understanding of oil extraction in the Amazon with a broader focus on economic, social, political, and health impact. Another six articles were excluded because they did not have specific data on cancer among indigenous people. The selection of studies is depicted with a flow diagram in Figure 1.

Of the 7 included studies, 5 reported results from cohort studies. All cohort studies were at a population level, with data on cancer burden retrieved from the National Cancer Registry or other government databases, and comparisons made between oil and non-oil producing provinces (except for one, comparing indigenous and non-indigenous people within oil-producing provinces). Four of them discussed all cancer sites in all ages, while one focused on childhood cancers. Measures of cancer burden and results were heterogenous and are discussed in the sections below. One study used a mixed-methods design to quantitatively estimate health risk and qualitatively explore perceptions of exposure to contamination by oil extraction. While the former was deemed acceptable according to US-EPA thresholds, low risk perception was reported to exacerbate indigenous people’s vulnerability to cancer. Finally, another study used a risk assessment method to study the concentration of metalloids in small scale farms of oil-producing provinces and estimate the associated risk to human health. High concentrations of certain carcinogenic metalloids were found, exceeding US-EPA thresholds.

### 3.1. Cancer Burden

Our search strategy yielded only one study clearly comparing the cancer burden of indigenous versus non-indigenous populations in the oil-exploited provinces of Sucumbios, Napo, Orellana, and Pastaza in eastern Ecuador. Together, they represent around 350,000 inhabitants, from which approximately 30% belongs to an indigenous group. This study of San Sebastian and Hurtig (2004) investigated cancer incidence in the Amazon Basin between 1985 and 2000, making use of the National Cancer Registry and ascribing indigenous ethnicity to those having two indigenous family names. A total of 1207 cases were reported, from which 48 were indigenous men and 62 indigenous women. In the latter groups, cancer of the testes (10.4%) and leukaemia (10.4%), followed by cancers of the penis (8.3%), stomach (8.3%), liver (6.3%), and lymph nodes (6.3%) were found to be the most common cancers among men, while cancer of the cervix uteri (22.6%), leukaemia (14.5%), skin melanoma (6.5%), stomach cancer, and colon cancer (both 4.8%) were most common among women. Across both genders, the age category between 15 and 44 was most affected. Comparing these numbers with the non-indigenous population, a lower cancer incidence of the stomach, skin, prostate, lymph nodes, and leukaemia in indigenous men was observed. In indigenous women, incidence of the stomach, skin, breast, cervix in situ, cervix uteri invasive and lymph node cancer was significantly lower [30].

This lower cancer incidence in indigenous people could be partly explained by their protective lifestyle, encompassing non-smoking status, healthy diet, physical activity, lower incidence of obesity, multiple and early pregnancies, and other factors [32]. However, other, and perhaps more important explanations relate to the possible underestimation of cancer burden in the indigenous population. First, cancer screening programs (e.g., cervical cancer screening) are very limited for the often isolated indigenous communities [33]. Secondly, there is no availability to histopathology and cancer diagnosis, except in the capital of Quito [34]. This implies that people who do not possess the resources to travel to Quito will not get a cancer diagnosis [30]. Thirdly, there are no mandatory cancer registries in the Amazon Region. Thus, in case of a clinical diagnosis of cancer, no record will be made in a cancer registry [34].

To summarize, the included articles tend to show a lower incidence of cancer in indigenous populations, but this might be attributed to several misleading factors. Moreover, different studies have indicated factors to which indigenous people are disproportionately exposed, leading us to believe they could even have a higher relative risk for cancer. These factors will now be discussed under the sections of social and environmental determinants.

### 3.2. The Impact of Oil Industry

Most available literature on determinants affecting indigenous health in Ecuador presumably concerns the oil industry, due to a long and vivid history of distrust among local communities [35]. While the International Agency for Research on Cancer (IARC) does not classify crude oil itself as a human carcinogen, several oil components and related activity products such as oil hydrocarbons (e.g., polycyclic aromatic hydrocarbons, benzene) and metalloids (e.g., chromium, cadmium) are carcinogenic. As one of many examples, benzene-polluted air or water has been shown to cause different types of leukemia through pathways of oxidative stress, genotoxicity, and immunosuppression [36,37,38]. Therefore, several studies have compared cancer burden in oil-exposed and non-oil-exposed regions.

Comparing cancer incidence according to residence near oil fields in the Amazon Basin, Hurtig, and San Sebastian (2002) found significantly elevated risk of stomach, rectum, skin melanoma, soft tissue, and kidney cancer in men; of cervix and lymph node cancer in women; and of haematopoietic cancers in children under 10 years of age [32]. Zooming in on the latter finding and extending the study period of 1985–1998 to 2000, a subsequent study from the same authors confirmed the significantly elevated risk of childhood leukaemia in oil-exposed counties [39].

It is true the associated burden depicted in the previous section merely reflects associations at a group level. Furthermore, errors in population estimates might exist. Still, several criteria are supportive of a causal relationship. These include the strength and consistency of the associations found in reliable studies, the plausible underlying biological mechanisms as well as the representative time sequence resulting from decades of oil pollution [32]. Considering the inconclusiveness of epidemiological studies confirming this hypothesis at the individual level, San Sebastian et al. (2001) have therefore recommended the implementation of environmental monitoring as well as a cancer surveillance system [40].

Finally, two studies used a different way to describe cancer burden in the indigenous population, retrieving cancer mortality from death certificates. Claiming the measure is less systematically biased than incidence, Kelsh et al. (2008) and Moolgavskar et al. (2014) compared the cancer mortality in provinces according to oil production. Both concluded they did not find any evidence of increased death in the Amazon provinces. Using such a hard endpoint however, this clearly underestimates the presence of an underlying disease, particularly for those cancers with a high survival rate. Moreover, a conflict of interest should be noted by the authors, as they were funded by the oil drilling company Chevron [34,41].

### 3.3. Interaction of Other Environmental and Social Determinants

Not all studies indicate a clear excess of carcinogenic risk related to oil activities. A study on drinking water quality in oil impacted regions for example, stated the carcinogen levels were inferior to (inter)national thresholds and acceptable for domestic use. Rather, they noted social determinants of exposure should be considered, such as socio-economic living conditions or vulnerability. Relying strongly on natural sources of water based on their ancestral culture, some indigenous communities prefer natural water from rivers or mountains. However, due to precarious living conditions, agricultural practices, and a lack of water treatment, significant water pollution can arise [35]. While Vargas et al. (2020) argue indigenous people are less informed about potential risks, Maurice et al. (2019) go even further by stating they tend to ignore these explanations [35,42]. Focusing the problem on oil companies would then be part of a symbolic process, resulting from the insufficient recognition about the oil exploitation they have suffered [35]. The importance of this difference in risk perception is also acknowledged by Barraza et al. (2018), adding to the debate that “estimates of exposure pathways … may be overestimated for people who are able to change their dietary and/or agricultural practices to limit their exposure, or underestimated in the case of persons who are socio-economically vulnerable and who cannot leave the impacted areas” [43].

A last point to raise is that oil activity is a possible but not exclusive source of exposure to carcinogenic compounds. For example, polycyclic aromatic hydrocarbons can also result from incomplete combustion of organic matter, vehicle exhausts, indoor cooking, or other work-related activities [32]. The same can be said for metalloid exposure, with geochemical bedrock composition, volcanic activities, deforestation, and widespread use of fertilizers being identified as additional sources. In the study of Barraza et al. (2018), high metalloid levels were found in most of the soils and some food products such as manioc or peach palm, respectively exceeding Ecuadorian and European Union Legislation limits. This is even more relevant in view of the fact that most indigenous people consume products they grow and harvest in their personal small-scale farms [43].

To summarize, there have been clear but inconsistent results associating oil-exposure in indigenous areas with cancer risk or even incidence. Moreover, even if the environmental magnitude is debated by some, we should bear in mind the exacerbating interplay with social determinants and other sources of carcinogenic compounds. Indeed, this double burden exposes the indigenous population to a particular risk.

## 4. Discussion

The present study explores the cancer burden among indigenous people and its links with oil exploitation in the region. By doing so this research seeks to consolidate evidence from a complex public health context with epidemiological studies putting forth starkly contrasting conclusions. This is a reflection of the ongoing protracted legal battle between the Chevron-Texaco oil companies and the local indigenous people. While civilian activists have focused on the health impact of commercial activities [40], studies funded by Chevron-Texaco have deflected such concerns and stressed the importance of sociological factors [35,42].

In keeping with a setting rife with contradictions, this study found contrasting accounts of the burden and determinants for cancer among indigenous Ecuadorians. Some studies minimized the health impact of oil activities in the Amazon, citing statistics of lower cancer burden among indigenous people and emphasizing the indigenous diet as a protection against any negative consequences. When explaining the rising prevalence of cancer among these communities, lack of awareness and socio-cultural norms were advanced as determining variables. Furthermore, some authors postulated that the infrastructure development which accompanies commercial activities could improve access to healthcare facilities [41]. However, it is important to note here that several articles espousing this theory had a conflict of interest, with authors receiving funding from oil companies. Articles bearing a potential conflict of interest have been indicated as such in the extraction table (Appendix A). This should be kept in mind when interpreting the results reported by these authors.

On the other side of the coin, a number of studies hypothesized that the occurrence of cancer is due to the environmental contamination carried out by commercial activities, a theory which has strong albeit inconclusive evidence. This can be better understood when viewed in the context of the Ecuadorian Amazon, where limited access to health centres impedes both detection and recording of cancer among indigenous people. It is also important to note the consequences of oil extraction in these regions, with several communities reporting unprecedented changes in their diet and lifestyle due to increasing modernization [44]. While commercialization of the Amazon has introduced road infrastructure, it has not yielded an improvement in educational or healthcare development [45].

Drawing together these different theoretical assumptions and the various research on this topic, we put forth three arguments to contextualize the occurrence of cancer and its determinants among the indigenous Ecuadorian people.

Our first argument is to emphasize the importance of broadening the epidemiological lens through which we view the health burden of cancer among these communities. Epidemiology as a data-driven science is inherently limited by its capacity for accurate data collection. The resource-constrained rural setting of Ecuador abounds with such limitations, where the lack of records on cancer incidence and mortality, insufficient cancer screening and diagnostic facilities, geographical inaccessibility of certain regions, and lack of healthcare infrastructure have compromised the strength of epidemiological studies [46]. Furthermore, socio-cultural factors are also at play with indigenous women often neglecting cervical cancer screenings due to embarrassment and rural-based communities unable to undertake the long, daunting journey to access elusive healthcare services in the city [47]. In such situations with data limitations, the scientific focus of epidemiology is most concerned with avoiding a type II error, i.e., claiming a relationship where none exists, and is consequently reluctant to make public health recommendations without conclusive findings [48]. However, waiting for sufficient evidence that can prove harm without a doubt is incompatible with the action-based orientation of epidemiology and its potential to meet public health needs. The precautionary principle has often been emphasized by popular epidemiologists, and most notably so in the context of the Ecuadorian indigenous communities [49]. Such a perspective would (i) allow us to consider the social factors that limit cancer diagnostics and recording when analysing data on cancer burden among this population, and (ii) consequently would allow us to shape public health policies that pre-empt or limit harm before it is too late to do so.

Our second argument focuses on an integrated view of various social and environmental determinants working in unison to impact health outcomes, particularly lifestyle-related diseases like cancer. Large-scale agricultural and oil companies have increasingly encroached on resource-rich Amazonian lands, displacing indigenous communities that inhabit these areas. In many cases, roads built to facilitate oil extraction were used to colonize the Ecuadorian Amazon for commercial agriculture [50]. This has culminated in mass displacement with only about 8% of the Oriente region belonging to indigenous communities by 1992 [51], and prompted migration to urban areas in pursuit of employment. Furthermore, oil extractive activities have resulted in the removal of natural vegetation and introduction of toxic materials such as petroleum, thus impacting the livelihoods of agro-based indigenous communities and necessitating adaptation in dietary patterns [52,53]. Thus, the spread of exploitative oil drilling and irresponsible waste disposal in the Amazon can indirectly cause negative health consequences such as cancer, by necessitating lifestyle changes among indigenous people. The social context of these indigenous groups can also exacerbate their exposure to environmental risk factors. Many Ecuadorian indigenous tribes live in areas with alarmingly high arsenic contamination in the water and soil and face heavy exposure through consumption of water and rice [54], but do not have the expertise or the financial means to improve their living conditions by relocating or changing their diet. Furthermore, their marginalized social status in Ecuador leaves them particularly vulnerable, with lack of cancer diagnostic and treatment services in rural areas and inequitable health access [30,33,34]. This illustrates that the various determinants associated with cancer do not act in a vacuum and underlines the importance of analysing potential social and environmental causes of cancer through an integrated framework.

Finally, we argue for a broader view of social determinants that encompasses macro-level factors beyond the individual’s immediate environment. While most research on health determinants among indigenous Ecuadorians centred on the role of oil companies or lifestyle changes, it would be hard to ignore the political and social climate that allow these factors to perpetuate. Studies decrying the damage inflicted by extractive commercial activities infer deficiencies of the state in safeguarding national resources, and the frequently lamented limitations to collecting epidemiological data speaks to a lack of public health investment. While multinational oil companies, such as the American-owned Texaco, followed strict standards for waste treatment and management in their home country, these restrictions were ignored in Ecuador. The lax environmental regulation and laws allowed them to continue damaging practices such as draining approximately 16 million gallons of polluted water into rivers, allowing seepage of waste into groundwater and abandoning pits filled with oil sludge [31]. Even after public backlash forced foreign companies to leave Ecuador, the state-controlled oil company PetroEcuador simply inherited and continued these exploitative practices [55]. The decades of social movements and bitter lawsuits for environmental justice, have been underlined by consistent political resistance to compromise on economic development. Despite the substantial political emphasis on development, there was a marked reluctance to develop the national health system, with healthcare services being largely privatized [56]. While significant progress was made under President Correa’s administration in doubling healthcare spending and developing universal health coverage, the benefits have not been equitably distributed and Indigenous communities have seen little changes in healthcare access and utilization [57,58].

Drawing together these perspectives, we offer suggestions for public health policy in Ecuador and further action-based research. We suggest particular attention to indigenous women, who are at the intersection of gender and ethnic inequities, which is reflected in the higher rates of cancer and lower rates of service utilization among this group [30]. To address this, we recommend interventions at different levels—informational campaigns to improve knowledge and change social norms, peer-delivered awareness to normalize treatment seeking and remove stigma, training of community lay-health workers (LHWs) to make cancer screening more accessible and extending the coverage of primary care services to improve cancer treatment for indigenous women. An additional focus of intervention that holds tremendous potential is promoting vaccination, namely HPV vaccination, to prevent cervical cancer, which accounts for the highest cancer burden among indigenous women. Considering the geographical remoteness and lack of primary care facilities in rural areas inhabited by indigenous people, it would be beneficial to turn to community-based solutions. An initiative that has been widely effective in other resource-constrained settings has been the training of community members to conduct screening and provide referrals. This has been particularly effective in overcoming stigma and normalizing treatment seeking and has significant potential among indigenous Ecuadorians.

Considering the prominence of socio-economic and environmental factors in determining cancer incidence and treatment, it would be important to use solutions that reconcile sustainable economic development with empowerment and protection of indigenous people. One such strategy focuses on involving indigenous people in commercial agriculture, and giving local communities greater control over development activities taking place in their territory [25]. An example of such a successful initiative is the Kichwa tribe who are legal guardians of about one million hectares of Amazonian land, and who’s eco-friendly agricultural practices have contributed to lower deforestation and improved financial empowerment of local communities [59].

There were some limitations to the scoping review process. Firstly, only English language articles and peer-reviewed scholarship were included. The language restriction acted as a limitation to including grey literature such as media reports and news articles, which are generally published in Spanish or local languages. There was also considerable variation in the types of measures used to evaluate cancer burden with some studies considering a diagnosis of cancer recorded in the national database as an estimate of burden while others used cancer mortality as an indicator. Additionally, no quality appraisal was conducted for the included studies as this is not considered essential to a scoping review, based on PRISMA guidelines [29]. In spite of these limitations, we remain confident that this review is a valuable contribution to the evidence base and provides an accurate scope of the current state of knowledge on cancer burden and its links to oil exploitation among indigenous Ecuadorians. By synthesizing diverse and often contradictory evidence, this review puts together a unified perspective of different determinants of cancer and advances recommendations for social and public health policy. Considering the phenomenon of resource extraction across the Latin American region, the recommendations from this study can also be considered in other similar contexts.

## 5. Conclusions

In conclusion, we would like to stress the importance of further research to strengthen the evidence of cancer and its determinants among indigenous Ecuadorians, while simultaneously initiating action-based research to put in place public health and social reforms. Such research and public health initiatives should be embedded within the community by empowering indigenous people and advocating for policy that invests in local resources.

## Figures and Tables

**Table 1:** Inclusion and exclusion criteria.

Selection Criterion	Inclusion	Exclusion
Population	Indigenous Ecuadorian people	General population, non-Ecuadorian population
Concept and Context	Main topic: Burden of cancerous diseases and neoplasms and its social and environmental determinants linked to oil exploitation	Main topic: not burden of cancerous diseases and neoplasms and its social and environmental determinants linked to oil exploitation
Language	English	Not English

**Table 2:** Search strategy.

Database	Search Strategy	Search Results
PubMed	(Ecuador) AND (Indigenous peoples[Mesh] OR Ecuadorian people OR Ecuadorian population OR native people OR tribal people OR first peoples OR aboriginal peoples OR autochthonous peoples) AND (Cancer OR tumor OR tumour OR neoplasms[Mesh])	125
Web of Science	TS = ((Ecuador) AND (Indigenous peoples OR Ecuadorian people OR Ecuadorian population OR native people OR tribal people OR first peoples OR aboriginal peoples OR autochthonous peoples) AND (Cancer OR tumor OR tumour OR neoplasms))	38
Google Scholar	(Social determinants OR environmental determinants) AND (Ecuador) AND (Amazonian OR Indigenous peoples OR native people OR tribal) AND (Cancer OR tumor OR tumour OR neoplasms)	3050

## Appendix A. Extraction Table

**Table A1:** Cancer burden and determinants among indigenous people in Ecuador.

Author (Year)	Title	Study Design	Study Population	Cancer Type(s)	Main Findings	Confounding Factors/Limitations	Conflicts of Interest
Hurtig (2002)	Geographical differences in cancer incidence in the Amazon Basin of Ecuador in relation to residence near oil fields.	Cohort	Data from National Cancer Registry for provinces of Sucumbios, Orellana, Napo and Pastaza, with 280,000 indigenous people and peasants. Within this population, comparison of oil-exposed counties compared to non-exposed ones	All	Relative risk (RR) of all cancer sites combined significantly elevated in exposed counties in men (RR = 1.40; 95% CI: 1.15–1.71) and women (RR = 1.63; 95% CI: 1.39–1.91). Significantly elevated risk of skin melanoma (RR = 10.15; 95% CI: 2.91–46.97), stomach- (RR = 2.51; 95% CI: 1.60–2.94), rectum- (RR = 10.40; 95% CI: 1.16–12.98), soft tissue- (RR = 15.59; 95% CI: 1.74–139.30) and kidney- (RR = 9.2; 95% CI: 1.03–82.20) cancer in men; of cervix- (RR = 4.01; 95% CI: 2.97–5.41) and lymph node- (RR = 4.74; 95% CI: 1.89–11.88) cancer in women; and of haematopoietic cancers in children under 10 years of age, both male (RR = 2.63; 95% CI: 0.90–7.69) and female (RR = 3.60; 95% CI: 0.95–13.57).	Possible underestimation due to lack of histopathological services in provincial hospitals, absence of cancer registry in the Amazon region, and difficulties of health care access due to geographics and socio-economic status. Ecological studies to be interpreted with caution (lack of data on individual exposure and outcome). Potential errors in population estimates (e.g., differential migration patterns) No information on (parents’) occupational exposures. Errors in methods ascribing ethnicity.	None
Hurtig (2004)	Incidence of Childhood Leukaemia and oil exploitation in the Amazon Basin of Ecuador.	Cohort	Data from National Cancer Registry for provinces of Sucumbios, Orellana, Napo and Pastaza, with 280,000 indigenous people and peasants. Extension of study period 1985–1998 to 1985–2000, with a reported total population of 356,406 indigenous people and peasants in the same provinces.	Childhood leukaemia and other pediatric cancers	A total of 91 reported cancer cases; 28 cases of leukaemia (RR = 2.56; 95% CI: 1.35–4.86; Incidence rate = 3.11) and 27 cases of other cancers (RR = 1.57; 95% CI: 0.90–2.76) identified in the exposed population, with significantly elevated relative risk in oil-exposed counties.		None
San Sebastian (2004)	Cancer among indigenous people in the Amazon Basin of Ecuador, 1985–2000.	Cohort	Data from National Cancer Registry for Provinces of Sucumbios, Napo, Orellana and Pastaza (29.7% of them indigenous, defined here as having two indigenous family names).	All	Indigenous men (RR = 0.26; 95% CI: 0.19–0.35) and women (RR = 0.23; 95% CI: 0.18–0.29) are generally at significantly lower risk of cancer compared to non-indigenous groups. Need to focus on factors explaining the different patterns in the future, as well as confounding factors.		None
Kelsh (2008)	Cancer mortality and oil production in the Amazon Region of Ecuador, 1990–2005.	Cohort	Mortality data with cause-of-death classifications for provinces of Napo, Orellana, Pastaza, and Sucumbios; compared to Pichincha province.	All	No evidence for increased cancer deaths in Amazon provinces. Unadjusted mortality rate ratios (RR = 0.82; 95% CI: 0.73–0.92) and age- and sex-adjusted mortality rate ratios (RR = 0.46; 95% CI: 0.43–0.49) for any cancer significantly lower compared to Pichincha.	Risk of aggregated exposure bias (ecological study). Cannot account fully for residential migration and hence period of exposure. Errors in death certificates (diagnostic errors, incompleteness, coding and processing errors, under-registration). cancer mortality rates will underestimate incidence, particularly for diseases with high survival rates.	Funded by Chevron
Mool- gavskar (2014)	Cancer mortality and quantitative oil production in the Amazon Region of Ecuador, 1990–2010.	Cohort	Comparison mortality data of 20 oil- and non oil-producing cantons in the provinces of Napo, Orellana, Pastaza and Sucumbios.	All	No evidence for increased cancer mortality deaths in oil-producing compared to non-oil-producing cantons. Poisson rate ratio (RR = 0.85; 95% CI: 0.72–1.00; *p*-value = 0.048) and standardized mortality ratios (SMR = 0.78; 95% CI: 0.72–0.84; *p*-value < 0.001) for all cancer-related deaths in the oil-producing cantons		Funded by Chevron
Barraza (2018)	Distribution, contents and health risk assessment of metal(loid)s in small-scale farms in the Ecuadorian Amazon: An insight into impacts of oil activities.	Risk assess-ment	Small-scale farms in the oil-producing provinces of Orellana and Sucumbios.	N.A.	Inhalation (79%) and water ingestion (19%) as the main exposure pathway for carcinogenic elements (mainly Arsenic and Chromium), exceeding US-EPA thresholds. Chromium accounting for 99% of the Total Cancer risk of the inhalation pathway.	High metalloid concentrations can also be explained by other natural (e.g., bedrock or volcanic ashes) and anthropogenic (e.g., agrochemical product use) phenomena Values should be balanced by the spatial heterogeneity of environmental contamination, as well as social factors (dependence on natural resources, knowledge of contamination risks and rights)	None
Maurice (2019)	Drinking water quality in areas impacted by oil activities in Ecuador: Associated health risks and social perception of human exposure.	Mixed method	Comparison of oil-producing and refining provinces of Orellana, Sucumbios and Esmeralda; to non-oil-producing regions of Morona-Santiago and Manabi	N.A.	Health index indicates acceptable chronic effects for domestic use of water according to the US-EPA thresholds.	Limits do not consider the cocktail effects of metallic and organic compounds.	None
